# Severity of Anxiety Disorders in Patients with Chronic Obstructive Pulmonary Disease

**Published:** 2015-04

**Authors:** Mitra Safa, Saeed Fallah Tafti, Firrouzeh Talischi, Fatemeh Ghassem Boroujerdi

**Affiliations:** 1 Clinical Tuberculosis and Epidemiology Research Center, National Research Institute of Tuberculosis and Lung Diseases , Masih Daneshvari Hospital, Shahid Beheshti University of Medical Sciences, Tehran, Iran; 2 Tobacco Prevention and Control Research Center, National Research Institute of Tuberculosis and Lung Diseases , Masih Daneshvari Hospital, Shahid Beheshti University of Medical Sciences,Tehran, Iran; 3 Nursing and Respiratory Health Management Research Center, National Research Institute of Tuberculosis and Lung Diseases, Masih Daneshvari Hospital, Shahid Beheshti University of Medical Sciences, Tehran, Iran; 4 Chronic Respiratory Disease Research Center, National Research Institute of Tuberculosis and Lung Diseases , Masih Daneshvari Hospital, Shahid Beheshti University of Medical Sciences, Tehran, Iran

**Keywords:** *Chronic Obstructive Pulmonary Disease*, *Anxiety*, *Depression*, *Hospitalization*, *Hamilton Questionnaire*

## Abstract

**Objective:** Patients with chronic physical diseases sometimes show increased loss of function; such patients need more care. Anxiety is a well-known symptom that is prevalent among chronic obstructive pulmonary disease patients that can prolong and increase the risk of hospitalization. The purpose of this study was to evaluate the severity of anxiety in the mentioned patients and to examine the presence of symptoms and appropriate treatment strategies to understand the role of psychological functions in physical patients.

**Methods** **:** This was a cross sectional study conducted in Masih Daneshvari Hospital. One hundred forty- three patients entered into the project by accessible method and signed the informed consent; they filled demographic information and Hamilton anxiety and depression questionnaires. Data were analyzed by SPSS-16.

**Results** **:** Of the participants, 68% were above 60 years of age; 78% were male; 89% were married; and 38% were self-employed. Also, among the participants, 51% were illiterate; 72% had history of smoking; 46% had history of substance abuse; and 49% had moderate to severe anxiety disorder. Moreover, of the patients with severe anxiety, 41.3% had severe muscle spasms; and severe sleeplessness was found in 38.5% of those with severe anxiety disorder. Severe anxiety related symptoms were found in 20.3% of the patients with severe anxiety disorder. Depressed mood was found in 27.3% of the patients with severe anxiety disorder. Severe physical and muscular signs were found in 35.7% of those with severe anxiety disorder.

**Conclusion** **:** According to our findings, many chronic diseases such as chronic obstructive pulmonary disease may contain anxiety and depression which result in vulnerability. Therefore, evaluation of anxiety in such patients is of importance for alleviating the disease.

Patients with medical illnesses are at risk of psychiatric disorders (1). Physiologic parameters of health and function in COPD patients interact with each other (2). Studies show that psychiatric wellness influences lung function response and pulmonary health (3). It is noteworthy to mention that patients with moderate psychiatric illness have shown worsening of their medical condition (4), and patients with co-occurrence of physical and psychiatric conditions show increased loss of function compared to having either conditions separately. These patients need more care and make emergency visits to hospitals more often. Additionally, psychiatric symptoms lead to decreased functioning, physical and psychiatric health (5).

Anxiety is a common psychiatric symptom seen in COPD patients even with improved breathing (as shown by FEV1 ) (6). It can also increase the length of exacerbation of illness and risk of re-hospitalization (7); and it is significantly more prevalent in COPD patients compared to the general population. After the exacerbation of the illness, 10 to 60% of the patients feel anxiety which stands out among other chronic respiratory conditions (8). Although the presence of anxiety and depression is high among the COPD patients, very few studies have been conducted on the health outcome of patients with symptoms of both COPD and psychiatric conditions. Psychiatric conditions are not regularly diagnosed in these patients and are not treated in a timely fashion (9). Awareness of the presence and severity of psychiatric disorders in patients with chronic pulmonary conditions encourages them to take the necessary measures for treatment. Psychiatric symptoms negatively influence disease severity and quality of life of the patients. Therefore, timely diagnosis and treatment are very important and delaying the treatment can delay recovery from the pulmonary symptoms and may cause complication (10).

Everybody experiences anxiety in his or her life, and it is natural to become anxious when facing a threatening life situation; yet, severe and chronic anxiety without a clear cause is abnormal.

Anxiety includes lack of assurance and physiologic triggering and sense of helplessness. It is an unhappy sensation, it a feeling of worry which co-occurs with such symptoms as fear, shortness of breath, palpitations, sweating and coldness of the extremities.

Symptoms and signs of anxiety include: nervousness, lack of calm, aggravation, tiredness, dizziness, polyuria, palpitations, weakness and shortness of breath, sweating, tremors, worry and fear, sleeplessness, difficulty concentrating and awaiting a fearful situation (11)

The Hamilton Anxiety Scale is a way to measure anxiety in individuals. It is a 14-item anxiety self-test, which gives a score on an anxiety scale; its correlation with Beck Questionnaire and SCL-90 is 0.6 and 0.73, respectively. The stability of the this questionnaire has been calculated to be 0.81 in Iran by test re-test method (16,12).

The purpose of this study was to evaluate the severity of anxiety disorder in COPD patients and to examine the increased awareness of the presence and severity of this disorder and to provide information about symptoms and timely and appropriate treatment.

## Material and Methods

This was a cross sectional study, with accessible sampling method conducted in 2012. In this study, 143 patients attending Massih Daneshvari Hospital were evaluated by internists’ spirometric interpretations and with definite diagnosis of COPD. They were referred to a psychiatrist and went through a mental status examination for the diagnosis of psychiatric disorder and signs of poisoning or withdrawal from abused substances based on DSM-IV TR criteria. If they were free of toxicity or withdrawal symptoms, upon their agreement and assuring them of privacy of the information, the patients completed the Hamilton anxiety and depression questionnaire in a calm and stress- free environment by an interviewer. 

The inclusion criteria included being older than 18, signing an informed consent, being conscious, not being toxicated by drugs or substances, no history of psychiatric diseases; and the exclusion criteria included being younger than 18, not signing the informed consent, having an impaired consciousness level, being toxicated by drugs or substances and history of psychiatric diseases. 

The Hamilton Anxiety Scale was used to measure anxiety. Demographic data were collected. Information was entered into the computer and analyzed with SPSS 16. To determine the correlation of anxiety with demographic variables, correlation analysis with significance level of 0.05was used.

The severity of anxiety was rated based on Hamilton scores as below:

<= 17: without anxiety, 18-24: mild, 25-30: mild to moderate, 31 and higher: moderate to severe (12 13)

In this study, only the severity of anxiety was measured without evaluation of the severity of COPD. 

Before beginning the study, ethical approval was obtained from the Research and Ethics Committee at the hospital, and all patients signed the informed consent to participate in the study. 

## Results

In total, 143 hospitalized COPD patients participated in the study. In this study, 6 individuals (4%) were between 20-39 years; 41 (29%) were between 40-59 years; 96(68%) were above 60 years of age. Of the participants, 32 individuals (22%) were female and 111 (78%) were male; 127 (89%) were married; 54 (38%) were self-employed; 40 (28%) were unemployed; and 26 (18%) were labor workers. Among the participants, 51% were illiterate and 3.5% had university education; 102 (72%) had history of smoking; and 66 patients (46%) had history of substance abuse (opium and others). Of the patients, 19.6% had mild anxiety disorder, 16.8% had mild to moderate and 49% had moderate to severe anxiety disorder. 

Among the patients with moderate to severe anxiety many were illiterate (53.4%); and of the patients with mild anxiety disorder many had university education (66.7%). Anxiety was not correlated with age or education (P>0.2).

Of the patients with severe anxiety, 41.3% had severe muscle spasms. On the other hand, among the patients with mild anxiety 9.1% had such spasms. Severity of anxiety and muscle spasms were significantly correlated (P<0.001).

Of the patients, 2.8% with mild anxiety had fears while severe fearfulness was found in 22.4% of the patients with severe anxiety. Severity of anxiety and fearfulness were significantly correlated (P<0.001).

**Table1:** Demographic Information

**Percent (%)**	
Male	78%
Female	22%
20-39 year olds	4%
40-59 years olds	29%
60 years of age and older	68%
Single and divorced	11%
Married	89%

**Table 2:** Employment and Education Status

**Percent (%)**	
Self-employed	38%
Unemployed	28%
Labor worker	18%
Illiterate	51%
University education	3.5%

**Table3:** Substance and Smoking

**Percent (%)**	
History of smoking	72%
History of substance abuse	46%

Severe sleeplessness was found in 4% of the patients with mild anxiety disorder while this sleeplessness was found in 9.8% of the patients with moderate anxiety and in 38.5% of those with severe anxiety disorder. Severity of anxiety and sleeplessness were significantly correlated (P<0.001).

Severe anxiety related symptoms were found in 1.4% of the patients with mild anxiety disorder, in 3.5% of the patients with moderate anxiety and in 20.3% of the patients with severe anxiety disorder. Severity of anxiety and related symptoms were correlated significantly (P<0.001).

Depressed mood was found in 2.8% of the patients with mild anxiety disorder, in 1.4% with moderate and in 27.3% with severe anxiety disorder. Severity of anxiety and presence of depressed mood were correlated significantly (P<0.001).

Severe physical and muscular signs were found in 4.2% of the patients with mild anxiety, in 5.6% of the patients with moderate and in 35.7% of those with severe anxiety disorder. Severity of anxiety and physical and muscular signs were significantly correlated (P<0.001).

## Discussion

Research in recent years has shown that non-communicable diseases can be associated with psychiatric changes in the body. Consequently, illnesses such as CNS disorder and cerebrovascular accidents, myocardial infarction, cancer, Parkinson's disease, hormonal imbalance and COPD may lead to anxiety and depression. As a result, patients become weak and do not receive care, so they are prone to exacerbations and increased impact of illness.

In this study, 19.6% of the patients had mild anxiety disorder, 16.8% had mild to moderate anxiety disorder, and 49% had moderate to severe anxiety disorder.

In our study, we have not considered the period of the disease, because the chronic physical patients are vulnerable to psychological symptoms like anxiety. 

The following studies are a review of research on anxiety disorders and severity among COPD patients:

In a study (Chronic Obstructive Pulmonary Disease, Anxiety and Depression: State of the Science), the research history of the disease was discussed from 2000 to 2009, and 75 articles on COPD, anxiety and depression had been published. They noted that the prevalence of anxiety and depression in COPD patients was higher than the general population. Additionally, patients were significantly influenced by their anxiety and depression. The latter conditions negatively influenced treatment of COPD, and patients were less compliant with the treatment and were more functionally impaired. Some studies showed that COPD patients with anxiety and depression disliked the stigma of psychiatric disorder and focused more on their physical symptoms rather than acknowledging their psychiatric problems. Authors of this study concluded that even though the prevalence of anxiety and depression among COPD patients is higher, serious barriers exist with regards to diagnosis and treatment of these co-existing conditions (13 14 ). In another study, Hospital Anxiety and Depression Scale were used to evaluate the prevalence of anxiety and characteristics in COPD patients. In that study, 93 patients completed the questionnaire at the beginning and were discharged from the hospital, and 50% had high scores at the beginning of hospitalization which decreased by the time of discharge. Anxiety was more prevalent among patients with history of anxiety and depression and among women (1).

In a study on psychiatric disorder among COPD patients, 118 hospitalized patients with COPD were recruited from 500 randomized samples of the general population and 500 psychiatric patients from the clinics who were assessed psychologically. It was shown that there was a significantly higher prevalence of psychiatric disorders, and particularly anxiety, in COPD patients compared to the general population, but this prevalence was less than what was observed in the psychiatric clinic patients. In this study, no correlation was found between the severity of COPD and anxiety (2).

In another study, COPD patients with severe anxiety or depression symptoms or both were compared with patients without such symptoms. A total of 202 COPD patients were evaluated for severity of anxiety, depression, pain and quality of life by completing the questionnaires. A sample of 114 patients was also elected as a control group for gender and health comparisons. The prevalence of anxiety and depression in COPD patients was 18.8% and 28.2%, respectively compared to 3.5% and 6.1% among controls. Female patients with COPD had higher severity of anxiety and depression and had poor quality of life. Illness influences health and quality of life which is dependent of various factors such as patients’ physical condition, expectations, coping skills and psychiatric well-being. Some studies have shown that psychiatric symptoms, particularly anxiety. May influence the patient’s health more than the severity of illness, other underlying medical conditions and demographic factors (5 (.

In addition, in another study, 828 patients completed the Beck depression inventory, anxiety questionnaire, psychiatric disorder questionnaire and critical care questionnaire. It was found that 96.4% had depression, 93.7% had anxiety and 97.7% had both. It was shown that depression, anxiety and panic disorder are common among COPD patients. 

In a study, 45 patients with severe emphysema were evaluated for co-presence of psychiatric disorder. A study was done using the Beck inventory and a panic and agoraphobia inventory; in this study, 44% of the participants had panic disorder and 40% had depression. Co-existent depression worsened panic symptoms (14,15). Another study has proved the association between COPD and symptoms of anxiety and depression. The severity of anxiety and depression were correlated with the severity of COPD and the presence of lower PaO2 (18, 16).

Substance abuse was also common among COPD patients. The most abused substances in COPD and asthmatic patients were cigarettes (78%) and opium (42%) (17). It was also revealed that the long term contact with toxins in cigarettes can lead to lung damage and alveolar dysfunction leading to emphysema and COPD. Acrolein and its derivatives are carcinogenic and can lead to chronic inflammation as in COPD (19,18). In a study in Iran, 29% had substance use history, and inhalational opium was the most common substance used (20, 19). Considering the co-presence of psychiatric disease, and a high noncompliance with medical treatment, behavior therapy found to be useful even for one session (15, 20).

**Limitations**

This study was limited to hospitalized COPD patients in a referral center for pulmonary patients, so a more generalized study with participation of the general population with COPD of less severity is highly recommended.

## Conclusions

It is obvious through the past studies that anxiety disorders are common among COPD patients. 

In this study, of the COPD patients, 143 were evaluated with regards to their psychiatric disorder via questionnaires and interviews. Anxiety and depression were more prevalent in the patients compared to the general population. Presence of depression was not correlated with severity of COPD. Near 8% of the patients were alcoholic; 5% were nonsmokers which had a prevalence of 62% among their spouses. Patients and their spouses had poorer quality of life. Considering the co-presence of psychiatric disease, and a high noncompliance with medical treatment behavior therapy found to be useful. 
